# A Direct Comparison of Alloderm-Ready to Use (RTU) and DermACELL in Immediate Breast Implant Reconstruction

**Published:** 2016-08-11

**Authors:** Michael R. Zenn, C. Andrew Salzberg

**Affiliations:** ^a^Duke University Medical Center, Durham, NC; ^b^Icahn School of Medicine, Mount Sinai Health System, New York

**Keywords:** ADM, breast reconstruction, breast implants, Alloderm, DermACELL

## Abstract

The objective of this study was to compare the 2 leading human acellular dermal matrices in breast reconstruction with implants. This retrospective study draws on the experience of 2 expert surgeons with a history of long-standing use of the Alloderm-RTU (LifeCell Corporation, Branchburg, NJ) product who switched to the DermACELL acellular dermal matrix (LifeNet Health, Virginia Beach, Va) product. The consecutive nature of these data over this change allowed comparison between the 2 products without the confounding effects of patient selection or change in technique. The postoperative complications of seroma, infection, implant loss, and unplanned return to the operating room were studied, and no statistical differences were noted between these 2 products. The overall complications rates were low, with implant loss and infection less than 2% in 249 cases. Recommendations are for continued use of acellular dermal matrix in breast reconstruction and product selection based on price and availability.

The use of acellular dermal matrix (ADM) is now accepted in immediate and delayed breast reconstruction.^1^^-^12 Its benefits derive from its soft-tissue support of the lower pole of implant breast reconstruction when used as a sling in conjunction with the pectoralis major muscle or in complete implant coverage in the prepectoral position. There is also some suggestion that ADM may protect against capsular contracture.^13^^-^16 All ADMs are not created equal and the products on the market vary in their composition and processing. The majority of the reports on use of ADM over the past decade have reported on one product, Alloderm, and most surgeons are familiar with its use. More recently, the number of ADM products in the market has increased and our experience with ADM has expanded. In the United States, human-based products are the most popular. The market leader, Alloderm RTM, launched in 1994 with the first reported use in breast reconstruction in 2004, has been the most widely reported upon. A newer product, Alloderm-Ready to Use (RTU), was launched in 2012. Although the products tend to be discussed in aggregation and are reported under the same HCPCS code, they are actually different products and data on the newer product are lacking. This article compares the newer Alloderm-RTU with another leading human ADM, DermACELL, which was launched in 2010, in a consecutive series of breast reconstructions and compares their effectiveness.

## METHODS

This study was approved by the local institutional review boards (IRBs) of Duke University Medical Center and Mt. Sinai Health System. After obtaining IRB approval at each of the authors’ institutions, a retrospective review of prospectively maintained databases of long-term ADM users (M.R.Z. and C.A.S.) was conducted to obtain a consecutive series of ADM-based breast reconstructions that spanned their use of Alloderm-RTU and DermACELL. Both had been long-term users of Alloderm and switched to DermACELL use during this study. The techniques used in reconstructing breasts did not differ between products. This allowed direct comparison of the 2 products in the hands of experienced surgeons without patient selection bias. Minimum follow-up for patients in this study was 6 months and ranged from 6 months to 2 years. Patient characteristics included age, indication for mastectomy (prophylactic, oncologic), immediate versus delayed reconstruction, and history of radiation or chemotherapy. The primary outcomes of complications evaluated included seroma, hematoma, capsule formation requiring surgery within 6 months, infection, and implant removal. Treatment of subsequent infections was recorded as treated with oral antibiotics or intravenous antibiotics.

## RESULTS

There were a total of 140 patients included in this retrospective review. These represent a consecutive series for both surgeons during their transition from Alloderm-RTU (70 patients) to DermACELL (70 patients). There was no statistical difference between the groups regarding age, indication (cancer vs prophylactic mastectomy), or the incidence of radiation or chemotherapy. The number of implants is not equal due to differences in these groups between the number of unilateral and bilateral cases (see Table 1).

The number of complications noted in this study was small (Table 2), with only 3 patients presenting with infections that cleared (2 requiring antibiotics by mouth alone and 1 requiring intravenous antibiotics), 1 patient with hematoma, no patients with seromas, and 12 patients requiring secondary unplanned surgery (1 for hematoma, 3 for explantation, 4 for skin necrosis, and 4 for early capsular contracture).

Statistical analysis of the data was performed using the Fisher exact test. First, differences between Alloderm-RTU and DermACELL from the standpoint of infection were explored. Using the data set of all expanders and implants (*N* = 249), the Fisher exact test *P* value (.7452) indicated that the association was not statistically significant at the .05 α level. Thus, we concluded that there was no relationship between ADM type and infection.

Next, the relationship between ADM type and need for surgery in the early postoperative period (first 6 months) was investigated. When using only the subset data of patients who had surgery (*N* = 12), the Fisher exact test *P* value (.5692) was greater than the significance at the .05 α level. Thus, we concluded that there was no relationship between ADM type and need for surgery in the early postoperative period. All 3 implant losses involved skin necrosis as the inciting factor and were not believed to be related to ADM type.

Other significant findings in review of the data showed an association between the infections and radiotherapy (*P* = .0045) and a lack of association between chemotherapy and infection (*P* = 1.0).

## DISCUSSION

Today's reconstructive surgeon faces many choices in ADMs for breast reconstruction. These can be divided by their source into human products, bovine or porcine products, or synthetic products. To differing degree, all can be effective but the best ADM in terms of effectiveness with the least complications has yet to be determined. Human products have been favored in the United States, whereas porcine products have been favored elsewhere. The market leader, Alloderm, a human product, created the market and has the most literature supporting its use. This literature, though, has been based on its older, freeze-dried, aseptically processed product that has recently been changed to a ready-to-use product that is terminally sterilized with a Sterility Assurance Level (SAL) of 10^−3^. The older data are not applicable to the newer product, and Alloderm-RTU is now on the same footing as other products of similar duration in the market. One such product, DermACELL (LifeNet Health, Virginia Beach, VA), has clinically been equally effective in our hands in breast reconstruction and prompted our inquiry as to which product has the lowest complication rate. DermACELL is also a human product, produced by LifeNet Health (Virginia Beach, Va), a 501(c)3 federally designated organ procurement agency, tissue bank, and medical device manufacturer. LifeNet Health has more than 32 years of experience supplying tissue allografts for a variety of surgical applications and is certified by the American Association of Tissue Banks. Ironically, LifeNet Health was once the donated tissue supplier for the older Alloderm RTM. The similarities between the products stop there, as each is processed differently to create the end matrix. DermACELL undergoes a proprietary Matracell processing that experimentally has demonstrated less residual DNA and better tissue ingrowth than Alloderm.^17^ More importantly, DermACELL undergoes a terminal sterilization process that makes the product sterile with an SAL of 10^−6^ usually required for implantable medical devices.^18^ Because ADM is not considered a medical device, Alloderm with an SAL of 10^−3^ is acceptable but leaves the risk of contamination in the manufacturing process at one in a thousand compared with DermACELL's risk of one in a million.^19^ It is this issue that prompted the authors to explore the use of DermACELL.

Meta-analyses of ADM in breast reconstruction have shown, when compared with the standard of muscle coverage without ADM, the incidence of infection increases as much as 4-fold.^10^ While this has been attributed to the technical aspects of the procedure and introduction of a new foreign body, recent experiences with sterile ADM products have demonstrated rates of infection lower than the standard seen in full muscle-only reconstruction (T. A. Pittman, et al., unpublished data, July 2016). This raises the question about the aseptic nature of the market leader as a potential source of this increase.

This study retrospectively reviewed the consecutive data of 2 experienced ADM users who had used Alloderm for more than a decade and had extensive experience with the newer Alloderm-RTU before utilizing DermACELL in this study. The consecutive nature of the data, spanning both products with no change in technique between products, has provided an opportunity to compare these products directly while controlling for surgeon expertise and selection bias. The data show that both products were equally effective for use in implant-based reconstruction.

Both Alloderm-RTU and DermACELL had low rates of infection and showed excellent incorporation in all cases except for the 3 cases of explantation. The need for surgery in the first 6 months was higher with Alloderm-RTU, but this did not reach statistical significance (Table 2).

This study has demonstrated that both Alloderm-RTU and DermACELL are effective in breast reconstruction with expanders or implants. Another important finding revealed in this review is that in the hands of experienced surgeons, rates of seroma and infection, the 2 complications most related to ADM type, can be lower than those seen with non-ADM reconstructions.^10^

An important implication of this study, given the equal effectiveness of these products with a comparable risk profile, is the potential cost savings to our health system. DermACELL is 15% to 35% less expensive in the US market for commonly used sizes. Centers such as ours that use DermACELL have realized thousands and thousands of dollars in savings over the study period.^20^

In conclusion, the paradigm shift that was created by the use of ADMs has changed the way that we practice implant-based reconstruction. This study has shown that there is no clinical difference between Alloderm-RTU and DermACELL and that there is no statistical difference between these ADMs in infection rate, implant loss, or need for corrective surgery within 6 months of placement. With all else being equal, based on generally accepted surgical principles, a sterile product will always be preferable to an aseptic product. With equal clinical performance between Alloderm-RTU and DermACELL, value-based care would dictate that the decision on which product to use will likely be made on nonclinical factors, such as availability and price.

## Figures and Tables

**Table 1:** Distribution of cases of ADM and the number of expanders versus implants*

	Total patients	Number of implants	TE	Gel implants
Alloderm-RTU	70	130	19	111
DermACELL	70	119	24	95
Total	140	249	43	206

*ADM indicates acellular dermal matrix; TE, tissue expander.

**Table 2:** Complications encountered by ADM type*

	Complication				
	Infection	Required surgery	Implant loss	Hematoma	Seroma
Alloderm-RTU	1 (0.8%)	7 (5.4%)	1 (0.8%)	0 (0%)	0 (0%)
DermACELL	2 (1.7%)	5 (4.2%)	2 (1.7%)	1 (0.8%)	0 (0%)

*ADM indicates acellular dermal matrix.
